# 3D meshes dataset of sagittal otoliths from red mullet in the Mediterranean Sea

**DOI:** 10.1038/s41597-024-03641-1

**Published:** 2024-07-23

**Authors:** Nicolas Andrialovanirina, Lauriane Poloni, Rémi Laffont, Émilie Poisson Caillault, Sébastien Couette, Kélig Mahé

**Affiliations:** 1 Univ. Littoral Côte d’Opale, UR 4491, LISIC, Calais, F-62100 France; 2 Ifremer, Unité HMMN, Laboratoires Ressources Halieutiques, 150 quai Gambetta, Boulogne-sur-Mer, 62321 France; 3 École Pratique des Hautes Études, PSL Université, Paris, 75014 France; 4 UMR CNRS 6282 Biogéosciences, Université de Bourgogne, 6 Bd Gabriel, Dijon, 21000 France

**Keywords:** Population dynamics, Biological techniques

## Abstract

This paper presents a dataset of 3D sagittal left otolith meshes from 339 individual red mullet (*Mullus barbatus*). These immature specimens were collected from 17 geographical areas covering the entire Mediterranean Sea. Measured biological parameters were: fish total length (TL ± 1 mm, range from 125 to 238 mm), total weight (W ± 0.1 g, range from 14.9 to 168.0 g), sex (S), sexual maturity staging (Mat). The 3D otolith dataset comprises high-resolution meshes of otoliths obtained using microtomography (29.2 μm voxel size). The data offer valuable insights into the morphological variability and population structure of red mullet populations in the Mediterranean Sea. Potential applications of the dataset include age determination, stock identification, and population connectivity analysis. These applications aim to enhance the understanding of red mullet populations and contribute to the sustainable management of marine resources in the Mediterranean Sea.

## Background & Summary

Among the numerous fish species inhabiting the Mediterranean Sea, the red mullet (*Mullus barbatus*) stands out as a primary commercial species supporting artisanal and industrial fisheries across this geographical area^1^. Understanding the dynamics and structure of red mullet populations is essential for sustainable fisheries management in the Mediterranean. The otolith contains valuable clues about the life history of individual fish and the environmental pressures they experience^2–5^.

The otolith, a incrementally grown calcified structure located in the inner-ear of fishes, plays a crucial role in hearing, balance and orientation^6^. It is found within otic sacs in a network of semicircular canals inside the ear. These otic sacs contain three pairs of otoliths known as *sagitta*, *lapillus*, and *lagena*^7^. The data presented in this paper pertains only to the sagittal otolith, which is generally the most voluminous and the most commonly studied in fisheries applications^2,4,5^. Otolith shape is influenced by a multifaceted interaction among environmental factors^8–18^, genetic inheritance^11,14,16^, and ontogenetic development^12,19–21^. Otoliths are metabolically inert (*i.e*., without post-deposition alteration or resorption)^6^, and their extraction and processing are less time-consuming and less expensive than other methods of stock delimitation (*e.g*., genetics)^2^. Therefore, it has become one of the most widely used proxies for age estimation, identifying stock boundaries, and population connectivity analysis of various fish species^2,5^.

Traditionally, otolith shape analysis has been conducted using two-dimensional (2D) images^5^, which offer only a limited perspective of the three-dimensional (3D) structure. This approach may introduce biases due to the object’s positioning during 2D image acquisition. Recent advancements in imaging technology have enabled the application of 3D shape analysis to otoliths, providing a more comprehensive understanding of their morphology. By capturing the whole 3D surface of otoliths in three dimensions, this method offers deeper insights into their structural complexity and biological significance.

3D analysis reveals features that are often overlooked in 2D analysis, thereby expanding our knowledge of otolith morphology. However, research on 3D otoliths has primarily focused on acquisition methods^22–26^ and understanding their function within the fish inner-ear^27–30^, rather than on aspects applied to shape analysis. A recent study using part of the present dataset from red mullet otoliths demonstrated the effectiveness of 3D analysis in addressing significant asymmetry originating from the inner-ear side, a phenomenon not observed in 2D analysis with the same otoliths^31^. Additionally, this 3D asymmetry also influenced the fish’s location, *i.e*. the intensity of the asymmetry varied depending on the fish’s environment. Consequently, this dataset on the 3D shape of fish otoliths represents a new opportunity for better understanding their morphogenesis processes. With the 3D meshes data, research could delve deeper into the relationship with fish growth (*i.e*. ontogeny evolution), genetic heritage, and the environmental parameters of the habitat, allowing for a more comprehensive exploration and understanding their impact of these factors on the otolith.

This paper presents a comprehensive dataset of 3D sagittal otolith meshes of red mullet collected from 17 locations from the Mediterranean Sea. The dataset includes high-resolution images obtained using micro-computed tomography (micro-CT or *μ* CT). Each otolith image is available with the individual biological parameters of each specimen. These measurements provide valuable information on the shape variability of red mullet otoliths, which can be used to infer age, assess growth rates, and identify stock structure. The creation of this dataset was motivated by the need for standardised, high-quality data to support research in fisheries science, marine ecology, and conservation biology. By providing researchers with access to a collection of 3D otolith models and associated biological fish data, this dataset facilitates studies on the population dynamics, connectivity, and resilience of red mullet populations in the Mediterranean Sea. Furthermore, the dataset has the potential for broader applications beyond red mullet research, serving as a valuable resource for comparative studies on otolith morphology and fish population dynamics across different species and geographic regions.

Overall, this dataset represents a valuable resource for researchers interested in studying the ecology, biology, and fisheries management of red mullet populations in the Mediterranean Sea. By facilitating access to 3D otolith data, this dataset contributes to the advancement of scientific knowledge and the development of evidence-based management strategies for sustainable fisheries in the region.

## Methods

### Fish sampling

**Fig. 1:**
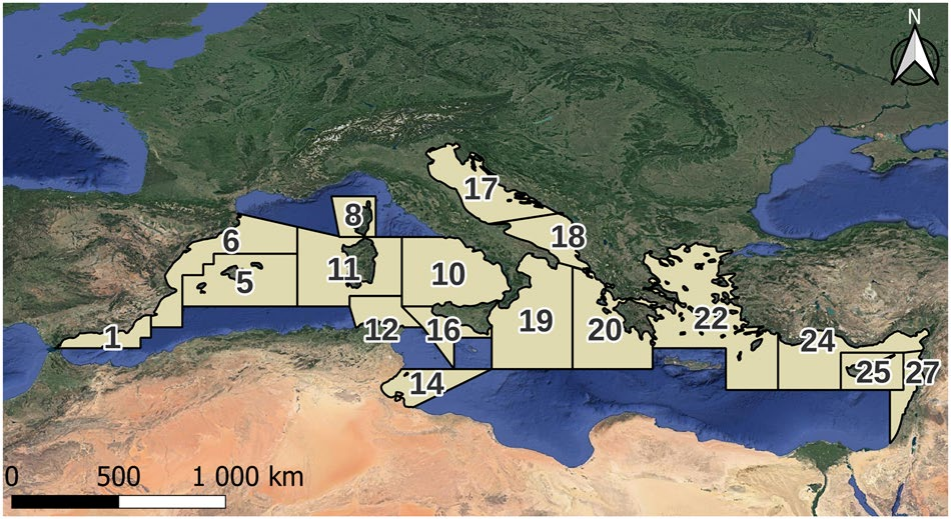
Location of fish sampling from 17 geographical sub-areas (GSAs) in the Mediterranean Sea.

339 individual fish specimens were collected from 17 geographical sub-areas (GSAs) (see Table 1 and Fig. 1), as defined by the General Fisheries Commission for the Mediterranean (GFCM). These GSAs represent diverse fisheries management unit within the Mediterranean Sea. The sampling campaign was conducted during the 2019 international MEDiterranean International Trawl Survey (MEDITS survey), a substantial effort to assess fish populations and their habitats in the Mediterranean Sea^32^. The MEDITS survey provides a valuable dataset for understanding the distribution and characteristics of fish species.

**Table 1:** Sampling distribution by geographical sub-areas (GSAs).

*n*^*o*^ GSA	1	5	6	8	10	11	12	14	16	17	18	20	22	23	24	25	27
n samples	7	23	12	12	13	5	19	16	58	8	30	20	36	19	22	24	27

To ensure robustness in analyse, potential ontogenetic effects on otolith shape are considered. Consequently, the sampling is restricted to young mature fishes (*i.e*. fish after their first sexual maturity and aged between 3 and 5 years old) within a total length range from 125 to 238 mm (with a mean length of 159 ± 19 mm), and a total weight range from 14.9 to 168.0 g (with a mean weight of 46 ± 19 g). Biological fish parameters and sample information were acquired (see Table 2). Furthermore, only left otoliths (*sagittae*) are selected to avoid asymmetric variation. These choices ensure uniformity in our dataset and facilitate accurate comparisons across individuals. Otoliths were carefully extracted from the inner ears of the fish, and cleaned to remove any adhering tissue or debris. Metadata describing the geographic sampling location, and the specimen characteristics (fish total length, weight, sex, maturity stage) were also obtained.

**Table 2:** Sampling details and biological fish parameters.

Parameters	Detail
Number	Sample identification number
Refscan	Unique sample reference
Species	Latin name of fish species
Meshes3D	3D meshes file name
Sdate	Fish catch date sampling (yyyy-mm-dd)
GSA	Fisheries management unit
Subarea	Geographical sub-area
Latitude	Latitude of the fish sample (in decimal degrees)
Longitude	Longitude of the fish sample (in decimal degrees)
Depth	Estimated depth of catch (in m)
BTemp	Bottom temperature (in celsius degrees)
TL	Total length of the fish, from the tip of the snout to the tip of the longest lobe of the caudal fin (in mm)
W	Total weight of the fish (in g)
S	Sex of the fish (M: male, F: female or I: undetermined)
Mat	Fish sexual maturity staging (0: abnormal *i.e*. necrotic, sclerotized or intersex gonads; 1: immature; 2: developing gonads, non-hydrated eggs; 3: spawning; 4: regressing or regenerating gonads; 5: no spawning)

### Three-dimensional otolith reconstruction

#### X-ray images acquisition

**Fig. 2:**
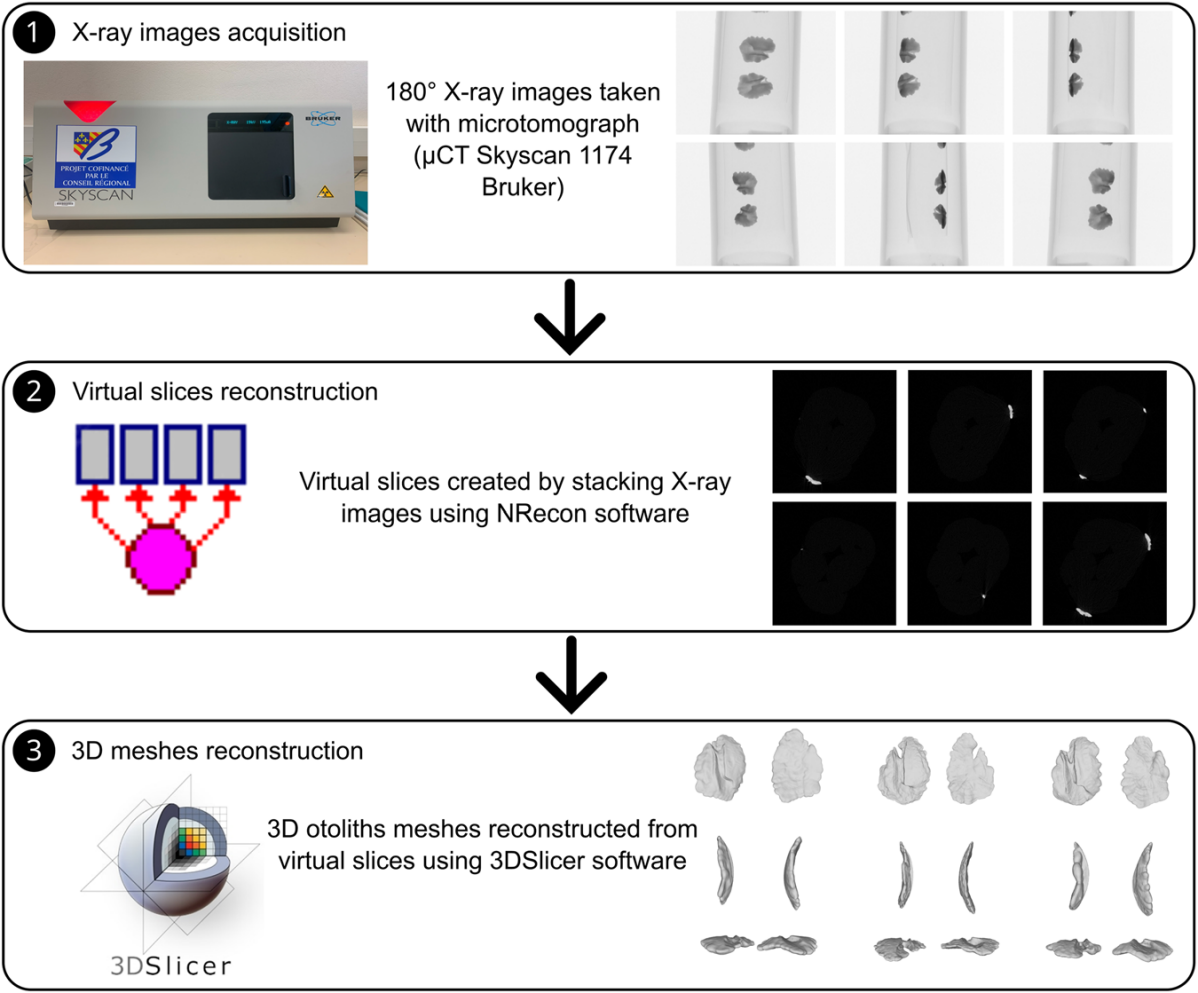
Method from X-ray images acquisition to 3D otolith meshes.

Otolith X-ray images were acquired using an X-ray microtomograph (micro-CT or *μ* CT) Skyscan 1174 (Bruker) (see Fig. 2). X-ray microtomography consists on entailed capturing two-dimensional (radiograph) images of the otoliths from various angles, covering a complete rotation from 0° to 180°. The differential attenuation of X-rays while crossing the sample depending of material density, and expressed as different grey values, enable us to discern quite easily the whole otolith from the surrounding air. X-ray microtomograph parameters are showed in Table 3.

**Table 3:** X-ray microtomograph parameters.

Parameters	Measures
Type of scanning	Oversize scan
Rotation step	by 0.7° (from 0° to 180°)
Number of catches	2
Exposure	3,000 ms
Filter	0.5 mm AL
Intensity	800 A
Tube voltage (tension)	50 kV
Voxel size	29.2 μm

#### Virtual slices reconstruction

The software NRecon by Bruker (v.1.7.4.6) was used to reconstruct cross-sectional slices of the otoliths from the radiographs acquired by the microtomograph (see Fig. 2). The program employs a Feldkamp algorithm, a convolution formula, and backprojection to reconstruct a three-dimensional density function from a set of two-dimensional projections. Indeed, X-ray images obtained by microtomography represent grayscale equivalences of material densities (higher densities will be encoded by higher grayscale levels, tending towards white). The algorithm compiles this set of images into a density volume and then generates slices in the transverse plane of this volume at regular intervals. This reconstruction step is automated and also allows for parameter optimization such as volume alignment, artifact correction, or selection of areas of interest. The slice images are saved in Tag Image File Format (.tiff).

#### 3D meshes reconstruction

**Fig. 3:**
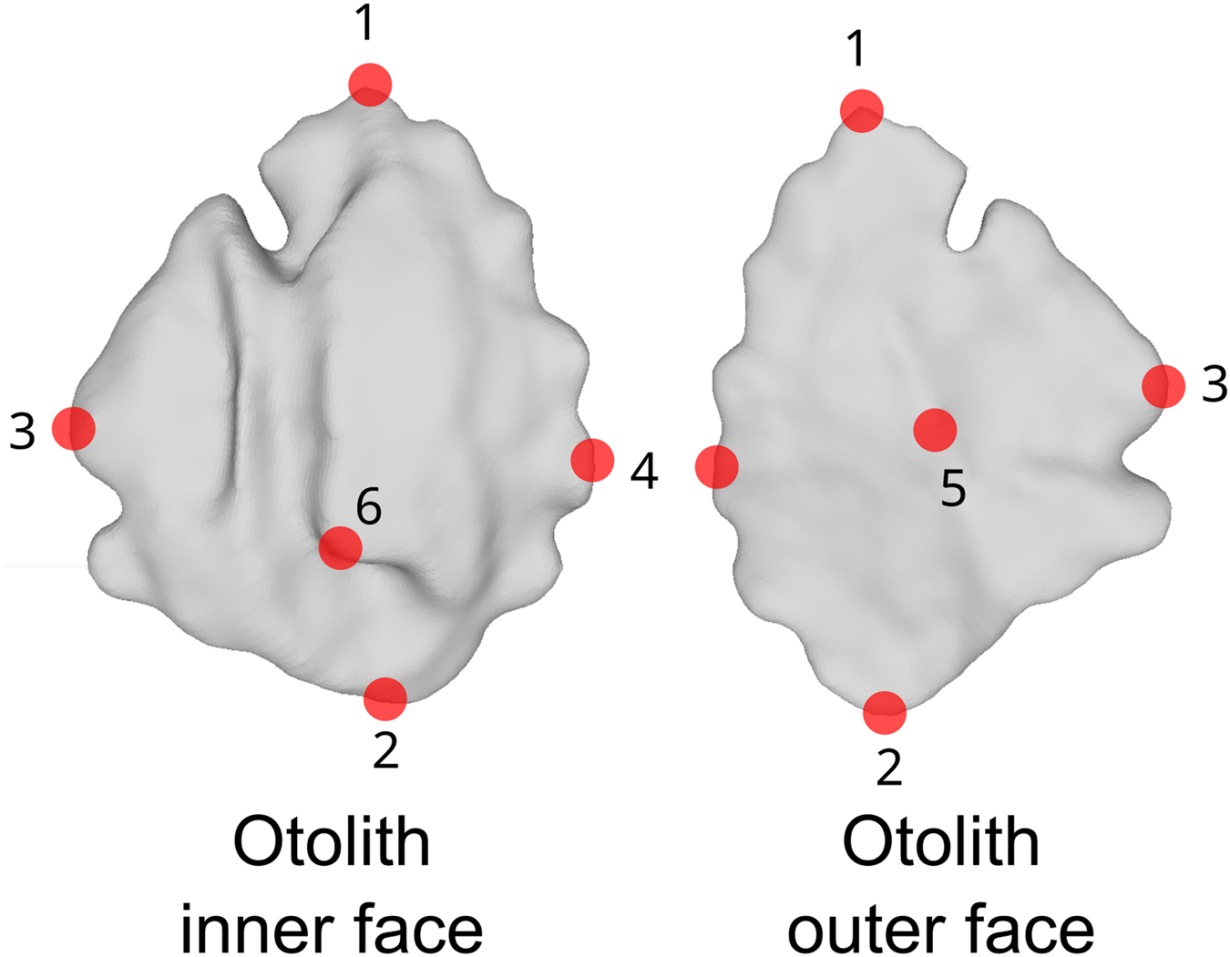
3D otolith meshes in inner and outer face with six landmarks (red points: 1 at the tip of the *rostrum*, 2 on the *postrostrum*, 3 at the end of dorsal, 4 at the tip of ventral, 5 on the middle of the outer face of the otolith, 6 in the transition part between *ostium* and *cauda*).

The segmentation step of the virtual slices aims to distinguish, within the resulting 3D volume of these slices, areas of interest corresponding to the objects under study (see Fig. 2). In this case, the goal is to differentiate between the voxels corresponding to the otoliths and the background voxels based on simple criteria of grayscale levels (the otolith being dense compared to the background, its constituent voxels will be bright) and adjacency (the otolith constituting a continuous object). This segmentation step is performed using the software 3DSlicer (v.5.0.2). Segmentation Editor module was used with threshold option. Threshold range was between 60 and 255. A grayscale voxel thresholding is applied to select all the voxels constituting the otoliths. The surface of this selected voxel set is then extracted in the form of a 3D mesh (see Fig. 2). This type of object models a 3D mesh as a cloud of points (vertices) connected by triangulation (see Fig. 2) expressing the surface of the 3D segmented object. 3D Slicer uses a Flying edges algorithm for the surface extraction and meshing. Landmarks were manually placed on the meshes to improve the alignment and measurement of the otoliths for future study. We proposed six landmarks (Fig. 3) placed on the meshes using the R package Digit3DLand^33^. These landmarks were chosen for their anatomical relevance and ease of identification across specimens, located at the anterior, posterior, dorsal, and ventral edges, as well as the core and a notable curvature point. These landmarks are only subjective, and we strongly recommend automatic alignment. These proposed landmarks could be used for pre-alignment before automatic alignment.

## Data Records

3D mesh data was saved in binary Polygon File Format (.ply). The x, y, and z coordinates of six landmarks (refer to Fig. 3) were stored in a TPS text file, where the ID corresponds to the mesh name. Sampling and biological fish parameters were registered in a single comma-separated value (.csv) file. In this csv data, each row corresponds to one fish, with 15 columns as detailed in Table 2. Log file after X-ray images acquisition (.log) and virtual slices reconstruction (_rec.log) were made available. In this way, all the information relating to image acquisition and reconstruction of the virtual slices was saved in these files if necessary. Readme text file is provided for information on the dataset.

All 3D meshes, tps file with landmarks, sampling and biological fish parameters on csv are available on SEANOE (10.17882/99980)^34^.

## Technical Validation

Most shape analysis tools, such as Fourier descriptors, generally require closed objects for processing 3D mesh data. The presence of holes in a 3D object prevents its contour from being analysed.

To address this issue, meshes with holes, non-manifolds (*i.e*., detached or split edges or vertices) or isolated elements (non otolith artefacts) needed to be identified. To identify 3D meshes with non-manifolds, MeshLab 2022.02 was used. In the following equation (1), genus in mathematic function with Euler characteristic^35^ were employed to identify manifold meshes with holes.

1 $$g=-1\times (0.5\times (V-E+F+b)-1)$$

Where *g* is the genus (number of holes), *V* is the number of vertices, *E* is the number of edges, *F* is the number of faces and *b* is the number of borders of the 3D meshes.

Subsequently, meshes with holes were either removed or reconstructed by readjusting the threshold range on 3DSlicer. Functions from Rvcg package^36^ were used to remove isolated artefacts.

## Usage Notes

The data^34^ provided in this paper offer valuable insights into the 3D shape of fish otoliths and can be utilized by researchers across various fields. It is advisable to normalise the data to ensure consistency across different datasets. Researchers may also consider performing additional processing steps, such as alignment, scaling, or feature extraction, depending on their specific research objectives. Landmarks could be used for these purposes. An R script could be supplied to standardise the 3D meshes with the landmarks (decimation, alignment, scale) and then to extract the Fourier coefficients using SPHARM-spherical harmonics^37^. In SPHARM analysis, landmarks can be used to ensure that the polarity “up-down", “right-left", and “front-back" is homologous among the meshes being compared.

### Supplementary information

Script related to 3D meshes dataset of sagittal otoliths from red mullet in the Mediterranean Sea

## Data Availability

Pre-processing code was written in R to delete non otolith artefacts, select meshes without holes, save the mesh as a .ply file, and place 6 landmarks for all 3D otolith meshes. The code is available in the supplementary information of this paper.
